# Computational Modeling Reveals Dendritic Origins of GABA_A_-Mediated Excitation in CA1 Pyramidal Neurons

**DOI:** 10.1371/journal.pone.0047250

**Published:** 2012-10-12

**Authors:** Naomi Lewin, Emre Aksay, Colleen E. Clancy

**Affiliations:** 1 Department of Physiology and Biophysics, Weill Medical College of Cornell University, New York, New York, United States of America; 2 Tri-Institutional MD-PhD Program, Physiology, Biophysics and Systems Biology Graduate Program, Department of Pharmacology, University of California Davis, Davis, California, United States of America; University of California Riverside, United States of America

## Abstract

GABA is the key inhibitory neurotransmitter in the adult central nervous system, but in some circumstances can lead to a paradoxical excitation that has been causally implicated in diverse pathologies from endocrine stress responses to diseases of excitability including neuropathic pain and temporal lobe epilepsy. We undertook a computational modeling approach to determine plausible ionic mechanisms of GABA_A_-dependent excitation in isolated post-synaptic CA1 hippocampal neurons because it may constitute a trigger for pathological synchronous epileptiform discharge. In particular, the interplay intracellular chloride accumulation via the GABA_A_ receptor and extracellular potassium accumulation via the K/Cl co-transporter KCC2 in promoting GABA_A_-mediated excitation is complex. Experimentally it is difficult to determine the ionic mechanisms of depolarizing current since potassium transients are challenging to isolate pharmacologically and much GABA signaling occurs in small, difficult to measure, dendritic compartments. To address this problem and determine plausible ionic mechanisms of GABA_A_-mediated excitation, we built a detailed biophysically realistic model of the CA1 pyramidal neuron that includes processes critical for ion homeostasis. Our results suggest that in dendritic compartments, but not in the somatic compartments, chloride buildup is sufficient to cause dramatic depolarization of the GABA_A_ reversal potential and dominating bicarbonate currents that provide a substantial current source to drive whole-cell depolarization. The model simulations predict that extracellular K^+^ transients can augment GABA_A_-mediated excitation, but not cause it. Our model also suggests the potential for GABA_A_-mediated excitation to promote network synchrony depending on interneuron synapse location - excitatory positive-feedback can occur when interneurons synapse onto distal dendritic compartments, while interneurons projecting to the perisomatic region will cause inhibition.

## Introduction

Despite widespread acceptance of GABA as the transmitter of inhibition in the central nervous system, there is plentiful evidence that GABA can also cause pathological *excitation* in the cortex. GABA_A_-mediated excitation has been suggested as a potential mechanism in diseases of neuronal hyperexcitability, such as epilepsy and neuropathic pain, and in neuroendocrine responses to stress [1]–[3]. Determining the mechanisms of GABA_A_-mediated excitation and its role in triggering pathological excitation may allow identification of new therapeutic targets to treat diseases of excitability.

In experimental slice preparations with glutamatergic blockade, synaptic activation of GABA_A_ receptors at frequencies that mimic rapid physiological firing leads to a reproducible cellular level phenomenon in isolated post-synaptic CA1 pyramidal neurons - paradoxical excitatory depolarization following the expected hyperpolarization [4]–[5]. This paradoxical depolarization is the apparent trigger that precedes neuronal firing and network synchrony in slices with intact glutamatergic transmission [6].

Although numerous experimental studies have attempted to reveal the underlying ionic mechanisms of GABA_A_-mediated excitation in CA1 pyramidal neurons, the precise contributions of intracellular chloride accumulation and extracellular potassium accumulation to depolarization remain unclear. Some experiments show substantial accumulation of intracellular chloride through the GABA_A_ receptor which causes depolarization of the GABA_A_ reversal potential (E_GABA(A)_) and may lead to GABA-mediated excitation [4], [7] [8]. Other experiments show substantial extracellular potassium accumulation in GABA-mediated excitation [9]–[10] [5]. It is critical to distinguish how intracellular chloride accumulation and increases in extracellular potassium contribute to GABA_A_-mediated excitation, since these levels are coupled through the primary chloride efflux mechanism, the potassium-chloride co-transporter, KCC2 [11] [7] [12] [5], which has been suggested as a potential therapeutic target to suppress pathological states in epilepsy and pain [13]. In order to understand how modulators of KCC2 activity should be used therapeutically, a clearer understanding of how potassium and chloride kinetics contribute to GABA_A_-mediated excitation is necessary.

To determine ionic mechanisms of GABA_A_-mediated excitation we have developed a biophysically detailed computational model of a CA1 pyramidal cell that accounts for the myriad processes contributing to ionic homeostasis. To our knowledge, no computationally based morphological neuron model yet exists that incorporates both chloride and potassium homeostasis mechanisms to allow for simultaneous study of these essential coupled (via KCC2) ionic subsystems [11] [7] [12] [5]. An advantage of the computational approach that we employ is to allow for testing of assumptions about the electrophysiological and ionic activity in dendrites that are generally based on recordings from the soma. Additionally, experimental factors that may lead to conflicting interpretations of experimental data, such as off-target effects by pharmacological agents, can be explicitly controlled in the computational model.

The model that we present suggests plausible ionic mechanisms of GABA_A_-mediated excitation and represents a foundation for future studies to investigate other disease mechanisms stemming from disruptions in ion homeostasis. The model may also be spatially extended to networks that can be used to investigate how GABA_A_-mediated depolarization in pyramidal cells triggers and supports sustained epileptiform activity in hippocampal circuits.

## Methods

### Model Reconstruction

**Figure 1:**
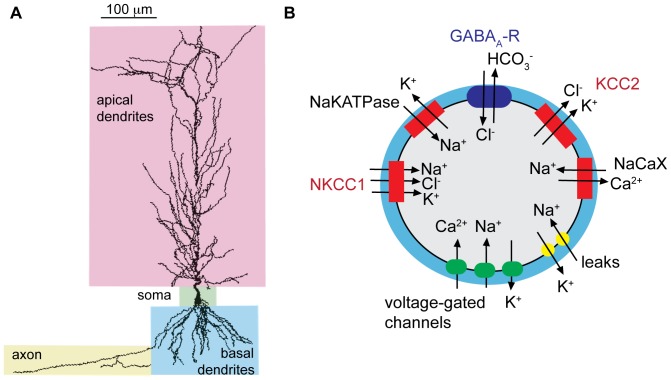
The anatomically based model of a CA1 pyramidal cell accounts for ion homeostasis. (A) The morphology of the model neuron is based on a CA1 pyramidal cell, with a soma, apical and basal dendrites, and axon as indicated. (B) Ion homeostasis is computed in each compartment by accounting for ion flux between the intracellular and extracellular space through channels, pumps and transporters, including voltage-gated Na^+^, K^+^, and Ca^2+^ channels, GABA_A_ receptors, neuronal K^+^−Cl^−^ cotransporter (KCC2), Na^+^−K^+^−Cl^−^ cotransporter (NKCC1), Na^+^−K^+^ ATPase (NaK-ATPase), the Na^+^−Ca^2+^ exchanger (NaCaX), and leak currents. The extracellular volume (blue shading in diagram) is 15% of the intracellular compartment.

The computationally based model neuron was morphologically reconstructed from hippocampal pyramidal neuron n123 taken from the published Duke Southhampton neuronal morphology http://www.compneuro.org/CDROM/nmorph/index/n123_t.html. The model was modified to include an axon, as described in Poirazi et al. [14] and shown in Figure 1A. The neuron contains 183 compartments. Voltage gated ionic currents were modeled as in Poirazi et al. [14] with some modifications as detailed below. The extracellular space was modeled as a cylindrical shell surrounding each compartment with a volume 15% of the intracellular compartment [15]–[16]. The initial intracellular concentrations of Na^+^, K^+^, Cl^−^, Ca^2+^, and HCO_3_^−^ were based on the intracellular and extracellular concentrations described in Smirnov et al. [10]. The concentrations of Na^+^, K^+^, Cl^−^, and Ca^2+^ were allowed to fluctuate in both the intracellular and extracellular compartments, except where specified.

### Numerical Method

The cable equation 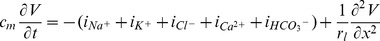, was numerically integrated using the implicit Euler method with time step 0.02 ms. Further decreases in time step did not change the model response. We also tested for convergence by reducing the spatial discretization unit. Doubling the number of compartments or quadrupling the number of compartments resulted in no qualitative change to the shape of depolarization/repolarization and resulted in a nominal change in the maximum voltage obtained in the simulation by ∼1 mV. We also tested the elimination of longitudinal diffusion, which also resulted in a nominal change to the maximal voltage by ∼1 mV.

The implicit Euler method was also used to update the state of the two currents described by Markov models, the GABA_A_ channel and fast sodium channel (described below). All other currents were updated using the explicit Euler method at every time step.

### Ion Concentrations

At each time step, intracellular and extracellular ion concentrations were calculated based on the sum of ionic current (from channels and transporters).
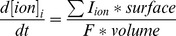
I_ion_ is the transmembrane current carried by each ion and F is the Faraday constant. Extracellular potassium concentration was buffered to represent glial buffering as in previous models [17]–[18]:



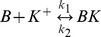


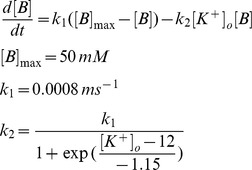



The model allowed for longitudinal diffusion of all ions between compartments with the following diffusion constants, in mm^2^/ms: D_Na_ = 1.33, D_K_ = 1.96, D_Cl_ = 2.08, D_Ca_ = 0.60 [19]. Longitudinal diffusion was calculated as in Kager et al. [20]:


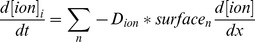
 where *surface_n_* is the connecting surface across which compartment *i* has a concentration gradient 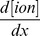.

### KCC2

**Figure 2:**
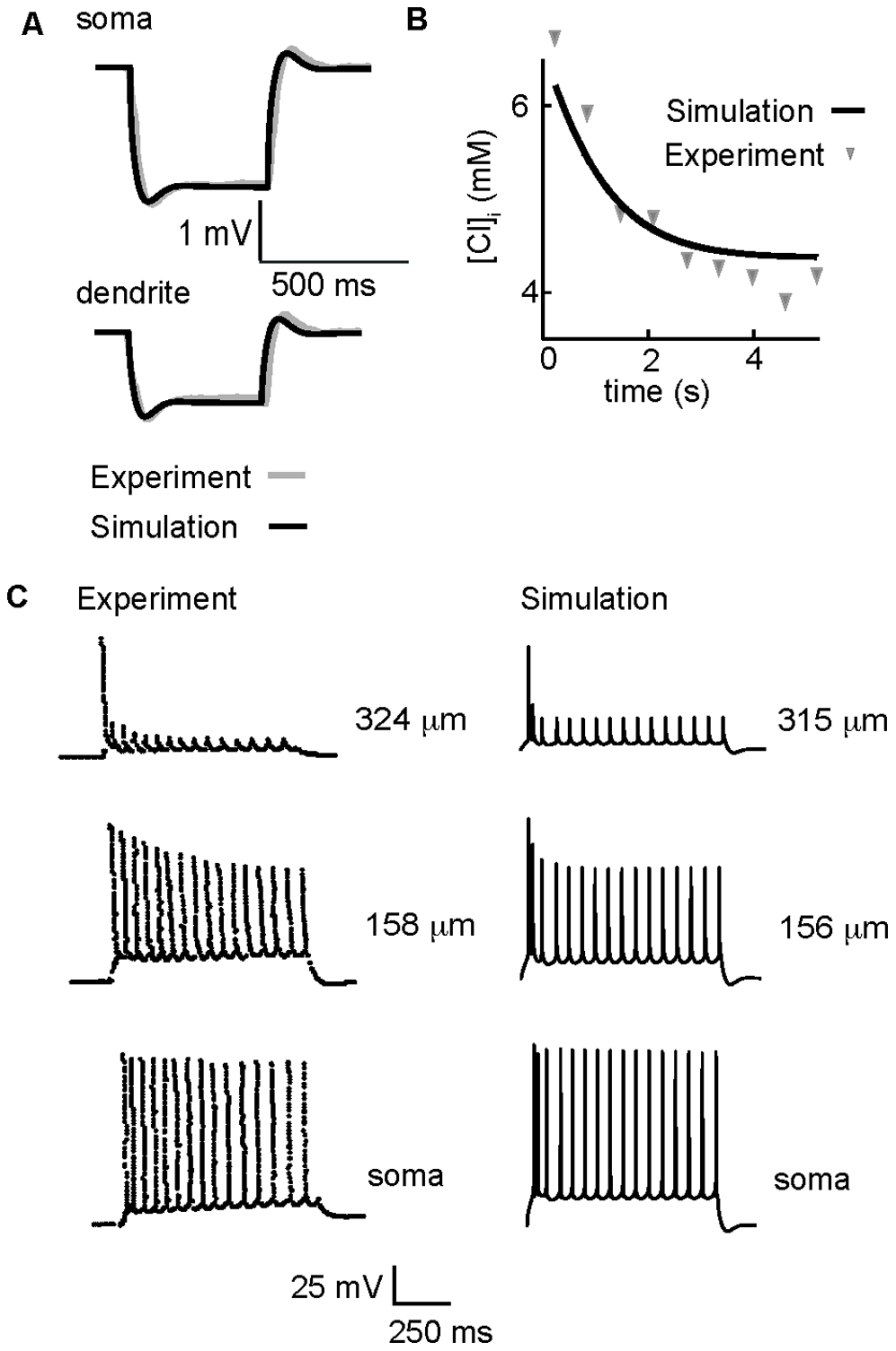
Tuning of model parameters based on experimental data. (A) Membrane potential elicited by somatic current injection as measured in the soma (A, top) and in a dendritic compartment 250 µm from the soma (A, bottom). The simulation (black line) is superimposed on experimental data from Golding et al. [34] (gray line). (B) Rate of intracellular chloride clearance in the dendrites. Intracellular chloride from an apical dendritic compartment 200 µm from soma (black line) is superimposed on experimental data [7] (symbols). (C) Action potential spike trains measured in the soma (bottom panels) and the proximal (middle) and distal (top) dendrites during 200 pA somatic current injection. Experiments are in left panels – simulations in right panels. Experimental data are from Spruston et al. [68].

The model for K^+^-Cl^-^ transporter KCC2 was based on a 2-state version of potassium-chloride cotransport previously developed to describe ion homeostasis in the renal distal tubule [21]. Measured affinities for K^+^ and Cl^-^ are from neuronal KCC2 in a heterologous expression system [12]. The transporter density used in our model was based on chloride clearance measurements from CA1 pyramidal cells as shown in Figure 2B [7].
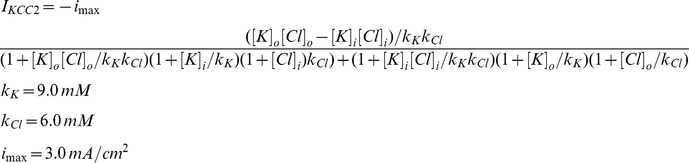



The current density carried by potassium ions transported by KCC2 is I_KCC2_ and the current density carried by chloride ions is –I_KCC2_.

### NKCC1

The NKCC1 model is from [22]. Flux via NKCC1 is given by:
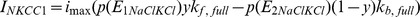
Where y represents the fraction of states on the external side and (1-y) the fraction on the internal side.



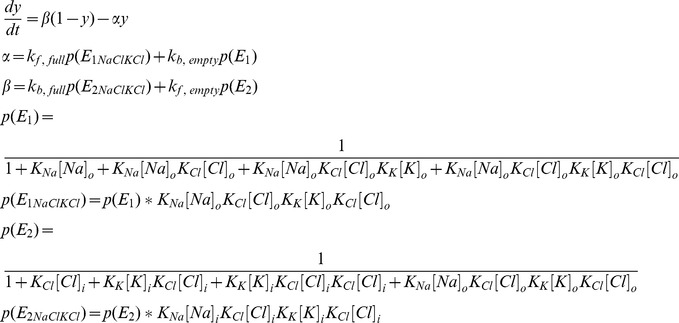



Where *p(E_1_)* represents the extracellular probability with no ions bound and *p(E_1NaClKCl_)* represents the extracellular probability with all ions bound, and *p(E_2_)* and *p(E_2NaClKCl_)* are the intracellular equivalents. The rates constants from Terashima et al. [22] are:
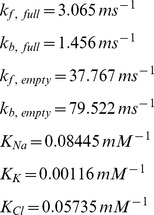



Sodium and potassium current is −.5*I_NKCC1_ and chloride current is *I_NKCC1_* due to transporter stoichiometry.

### Calcium Pump and NaCaX

The calcium pump is based on calcium-dependent extrusion rates from Kager et al [23]:
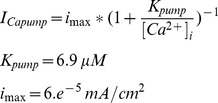



The Na/Ca exchanger model is from previous models of calcium dynamics in atrial cells and neurons [23], [24]:
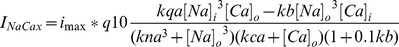


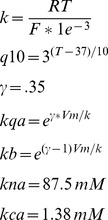



The sodium current transporter by the exchanger is 3*I_NaCaX_ and the calcium current in −2*I_NaCaX_. *i_max_* is set to balance calcium pump and calcium currents at rest.

### Voltage-gated Channels

The voltage-gated currents, with the exception of the delayed rectifier potassium current and the fast sodium current as described below, are detailed in Poirazi et al. [14].

#### Delayed rectifier potassium current

Voltage-dependent activation and inactivation from Gasparini et al. [25]:
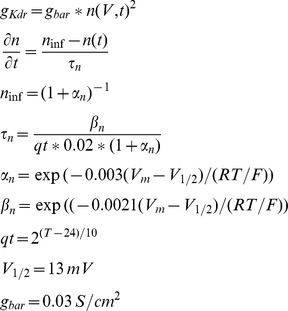



#### Fast sodium current

We incorporated a five state Markov model of fast sodium current that lacks slow inactivation and a six state Markov model that contains slow inactivation developed by Menon et al. [26]. These models were shown to accurately reproduce activity- and distance-dependent action potential attenuation in CA1 cells.

Following Menon et al. [26], only the model lacking slow inactivation was present in the soma. The fraction of sodium current with slow inactivation intact then increased linearly with distance to 250 µm from the soma (the total density of sodium current was constant). At distances greater than 250 µm from the soma, only the slowly inactivating component of current was present.

Total fast sodium conductance densities were as follows: in the axon, *g_bar_* = 0.1 S/cm^2^; in the soma and dendrites with radius >0.6 µm *g_bar_* = 0.014 S/cm^2^, and in the smaller dendrites *g_ba_*_r_ = 0.0014 S/cm^2^. The fraction of the sodium current carried by the 5-state and 6-state channels was: *fraction* = (distance from soma in µm)/250 if distance <250 and *fraction* = 1 if distance >250. Therefore, sodium channel conductance is calculated:
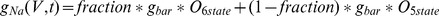
where O is the percent of sodium channels in the open state in the six state (O_6state_) or five state (O_5state_) model.

### NaKATPase and Leak Currents

The Na/K pump was based on Kager et al. [20]:
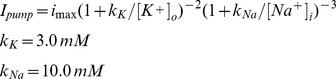
where ion flux in mA/cm^2^ is *I_K,ATPase_*_ = _−2*I_pump_ and *I_Na,ATPase_* = 3*I_pump_. i_max_ was determined by the model algorithm to be 0.0024 mA/cm^2^ in the soma.

The resting membrane potential of the model neuron is assumed to be constant at steady-state, and so requires balanced ionic homeostasis. The minimum NaKATPase current density at rest must balance sodium and potassium flux via the voltage gated currents and ion transporters described above. We set the NaKATPase current to be twenty percent above this minimum in order to have a sufficient potassium leak to allow the cell to respond to extracellular potassium transients by depolarization, as observed experimentally. Sodium and potassium leak current amplitudes were the final parameters determined in the model, set at rest (−66 mV) to balance ion flux from voltage-gated channels and ion transporters. This led to both a membrane resistance and responses to extracellular potassium transients that fell within experimental values: the membrane resistance is 53 MΩ (see Figure 1A), and the model responds to a 4 mM increase in extracellular potassium with a 3.9 mV increase in steady-state membrane potential (experimental values reported between 2.4 and 5.0 mV [27], [28]).

### GABA_A_ Synapses and Distribution

GABA_A_ current was modeled using a 6-state Markov model as was done previously by Bai et al. [29] with two binding steps for the GABA transmitter and two desensitization states [29]. The GABA_A_ current was carried by chloride and bicarbonate ions, based on measurements of a bicarbonate:chloride permeability ratio of 1∶4 of GABA_A_ channels [30]. Each synapse had a quantal conductance density of 1 nS, estimated from experimental data [31].

#### GABA_A_ synapse

Rate constants were taken from the α1β2γ3 receptor rates in Bai et al. [29].
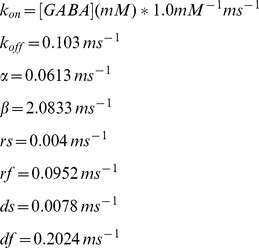



Synaptic density is in Table 1.

**Table 1:** Synaptic density was fixed to reflect somatic and dendritic distribution of GABA_A_ synapses observed in immunohistochemical labeling in CA1 pyramidal neurons [32].

Compartment type:	GABA_A_ density (synapses per µm length):
Soma, proximal apical dendrites (<150 µm from soma)	1.7
Radiatum/thick/medial	0.5
Radiatum/thick/distal and radiatum/thin	0.15
Lacunosum-moleculare	0.12
Oriens, proximal	0.61
Oriens, distal	0.1

### High-frequency Stimulation

To simulate high-frequency electrode stimulation of interneurons in *in vitro* slice preparation experiments, we modeled brief square pulses of GABA transmitter with a maximum concentration of 1 mM for 1 ms, and an exponential decay time constant of 100 microseconds based on experimental evidence [33]. Unless otherwise noted, the GABA pulses were applied both to the soma and apical dendrites to mimic stimulation of interneurons in the *stratum radiatum* of CA1.

### Simulations

Simulation of “voltage clamp” experiments assumed that only somatic compartments were clamped; voltage in the dendrites were not held constant, based on empirical evidence that voltage clamp in CA1 pyramidal neurons is imperfect and that there is significant attenuation of voltage in the dendritic tree [34]. Simulations were performed using an implicit Euler method with a timestep of 0.02 ms; further reduction of the time step did not change results. All source code was written in C/C++ programming language. Simulations were run on a Sun Fire X4440 x64 Server and multiple Apple Intel based Mac Pros 3.0 GHz 8-Core using OpenMP with the Intel ICC compiler version 11.1. Numerical results were visualized using MATLAB R2009a by The Math Works, Inc. All source code used in this study will be made available on ModelDB and is available by request by emailing ceclancy@ucdavis.edu.

## Results

### A CA1 Pyramidal Cell Model that Accounts for Ion Homeostasis

In order to better understand the underlying ionic mechanisms of GABA_A_-mediated depolarization in post-synaptic CA1 pyramidal cells, we developed a computational model that incorporates components critical for ion transport to account for electrical activity in the cell, and importantly, for ion homeostasis. The morphology of the CA1 pyramidal neuron is derived from geometry of a real neuron extracted from cell n123 in the hippocampal neuron database as described in the Methods and shown in Figure 1A. The model comprises a reconstruction of this geometry with 183 discrete compartments.

Detailed descriptions of all ionic processes are in the Methods and depicted schematically in Figure 1B. Briefly, Na^+^ homeostasis is maintained by influx via the voltage-gated Na^+^ channel, a leak current, and the sodium-calcium exchanger NaCaX; efflux occurs via the sodium-potassium pump NaKATPase and the sodium-dependent potassium-chloride transporter NKCC1. K^+^ homeostasis is preserved by efflux via voltage-gated channels, a K^+^ leak current, and the potassium chloride transporter KCC2, and influx via the Na-K ATPase and NKCC1. Cl^-^ homeostasis is maintained by influx via GABA_A_ channels and NKCC1 and efflux via KCC2. Ca^2+^ homeostasis is maintained by influx via voltage-gated channels and efflux via NaCaX and the membrane calcium pump. The intracellular and extracellular bicarbonate concentrations are assumed constant based on experimental data [7]. The extracellular space surrounding each compartment incorporated potassium buffering to represent the function of glial cells. The extracellular volume was 15% of the intracellular compartment (depicted in blue) [15]–[16].

### Model Fitting to Key Experimental Data

We next used experimental data that describe functional properties stemming from ion flux, ion channel kinetics and ion channel densities in CA1 pyramidal cells to guide parameter estimation of key components in our model. Figure 2A shows voltage traces recorded experimentally (grey line) and simulated (black line) in the soma (A, top) and a dendrite 240 µm from the soma (A, bottom) elicited in response to 50 pA subthreshold hyperpolarizing current injected in the soma. The timecourse and amplitude of the membrane voltage reflects activation of leak currents and transporters (see detailed description of the process used to tune these parameters in the Methods) and one active conductance - the hyperpolarization-activated mixed cation current (I_h_) (M-current is active in the model during the subthreshold stimulation, but is 17-fold smaller than the leak currents and has negligible contribution to determining the membrane potential). I_h_ density was adjusted to match experimental input resistance and voltage attenuation observed in the dendrites (bottom compared to top panel in A). The model has a somatic input resistance of 53 MΩ, compared to experimental values of 54+/−2 MΩ [34]. The simulated voltage traces match the amplitude and timecourse of the experimentally measured responses from an *in vitro* CA1 pyramidal neuron [34] and with the empirically chosen parameters, the model accurately reproduces a blunted voltage response in the dendrite compared to the soma.

In order to properly account for homeostasis of K^+^ and Cl^−^, we incorporated a model of the dominant K^+^-coupled Cl^−^ extrusion mechanism in CA1 cells as suggested by experiments, the neuronal K^+^-Cl^–^ cotransporter (KCC2) [35]–[36] [13] [37]. KCC2 maximum transport density was empirically tuned to reproduce the timecourse of experimentally measured kinetics of chloride extrusion [7] as shown in Figure 2B.

A notable feature of CA1 pyramidal cells is activity-dependent attenuation of back-propagating action potentials [34]. Attenuation in action potential (AP) amplitude increases with distance from the soma as shown in the Figure 2C, left. Note the increasing attenuation reported in the proximal and distal dendrites compared to the soma (from [34]).

Experiments suggest that attenuation of back-propagating action potentials results from an increasing fraction of slow cumulative Na^+^ channel inactivation in the dendrites [38]–[40]. Thus, using previously developed models [26], we incorporated two fast sodium currents, one with slow inactivation and one without slow inactivation. These models were shown to accurately reproduce activity-dependent dependent attenuation of back-propagating action potentials in CA1 cells. In the soma only the sodium model lacking slow inactivation was present [26]. A gradient in fast- versus slow-inactivating sodium channels was applied along the model dendritic tree and empirically tuned to reproduce experimental timecourse of action potential firing and amplitude of attenuation (described in detail in Methods). The presence of slow inactivation results in a loss of channel availability, and consequently current, with repetitive stimulation. The presence of fast and slow inactivating Na currents is sufficient to reproduce the attenuation and frequency of action potential spiking observed experimentally in various locations in CA1 pyramidal cells (Figure 2C, right). In response to a one second somatic injection of 200 pA, the model neuron fires at 15 Hz, the same rate as the experimental recordings.

Figures 1 and 2 summarize the construction of a computational model of the CA1 pyramidal cell with the experimental data used to empirically tune the model parameters that are critical for observed electrical behavior at rest, in response to stimulation, and the primary chloride extrusion kinetics. Next, we wanted to ask whether this model – *without any further tuning of parameters* - was sufficient to reproduce experimentally observed post-synaptic GABA_A_-mediated depolarization in CA1 cells following high frequency stimulation of synaptic GABA_A_ receptors.

### GABA-mediated Excitation: Model Validation

**Figure 3:**
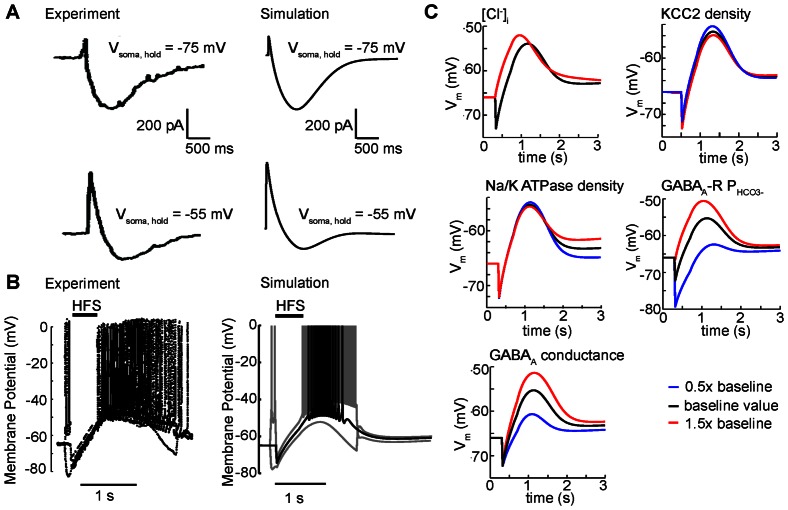
High-frequency GABA_A_ stimulation causes paradoxical depolarization in CA1 neurons. (A) Experimental and simulated currents recorded in the soma under voltage clamp at −75 mV and −55 mV. Simulations (right panels) are compared to experimental data (left panels) from Smirnov et al. [10]. Stimulation of 40 pulses at 100 Hz was applied (50% activation of GABA_A_ synapses in the simulation). (B) Somatic membrane potential in experiment from Viitanen et al. [5] (left) and simulation (right) (80% activation of GABA_A_ synapses in the simulation). In both the experiment and simulation identical HFS in presence of a transient 200 pA depolarizing or hyperpolarizing injection current in the soma did not change the trend for membrane potential in the soma to tightly follow the GABA reversal potential in the soma during stimulation. (C) Coarse sensitivity analysis demonstrates the robustness of the GABA_A_ mediated depolarization to substantial changes (red indicates 50% increase in parameters as indicated. Blue indicates 50% reduction in indicated parameter) in baseline model parameters (without sodium current). See Methods for the baseline parameter values.

To determine whether our model had the elements necessary to reproduce the GABA_A_-mediated excitation observed experimentally, we applied high frequency GABA pulses in the model to mimic the stimulation protocol in a semi-intact network as in experiments (glutamatergic and GABA_B_ synapses blocked): 40 pulses at 100 Hz were applied to the soma and apical dendrites in the model neuron (applied to *stratum radiatum* in experiments) [9]–[10] [5]. We compared the model-generated currents to experimentally recorded currents under identical conditions of voltage clamp (Figure 3A). Activation of 50% of the maximum GABA_A_ synaptic conductance in the soma and apical dendrites in the model neuron (to simulate a barrage of interneuron activity) during each pulse led to currents with similar time-course and kinetics to those observed experimentally under voltage clamp conditions at −75 mV and −55 mV (Figure 3A, top and bottom). Experimentally, a large network of interneurons is activated by the high frequency stimulus (HFS), which is accounted for in the model by the assumption that a high percent of the total GABA_A_ synapses are activated, as would be the case in a barrage of interneuron network activity (rather than a few isolated GABA_A_ synapses as would occur if a single interneuron was activated). Note that the precise magnitude and distribution of the stimulation of GABA_A_ receptors is unknown and will vary from cell to cell. Hyperpolarizing and depolarizing currents of the magnitude observed experimentally were achieved in the model by stimulation of GABA_A_ conductance that was within the experimental estimates of GABA_A_ conductance in CA1 pyramidal cells [31]–[32].

Next, we compared experimentally recorded and model generated voltage traces under current clamp conditions (Figure 3B). An 80% stimulation of the maximum GABA_A_ receptor conductance in the apical dendrites during high frequency stimulation led to membrane potential measurements comparable to those observed experimentally (Figure 3B, black lines in both). In both the experiment and model generated voltage traces, HFS results in rapid hyperpolarization of the membrane potential followed by a slow persistent depolarization that brings the membrane to threshold for AP generation, resulting first in a burst of APs and then slow repolarization. We also mimicked additional experimental conditions [5] by applying identical HFS in the presence of either a 200 pA hyperpolarizing or depolarizing current injection to the soma for 2 seconds (Figure 3B, gray lines). The experiment demonstrated that during stimulation there is sufficient GABA conductance to clamp the membrane potential to the GABA reversal potential [5]. In our model, as in the experiment, the membrane potential in the soma is strongly tied to GABA_A_ reversal potential in the soma during the stimulus.

In order to be sure that the model-generated GABA_A_-mediated depolarization was not overly dependent on our particular choice of model parameters, we conducted a coarse sensitivity analysis to test the robustness of GABA_A_-mediated depolarization to perturbations in key parameters. We asked whether the GABA_A_-mediated depolarization persisted when large changes to key parameters were applied, since a range of experimentally reported values or a paucity of experimental measurements limit our confidence in precise values for some model parameters, including intracellular chloride concentration, KCC2 transport dynamics, extracellular potassium buffering capacity, GABA_A_ bicarbonate permeability, and GABA_A_ conductance density [30] [41] [37] [31] [42]–[44]. We therefore examined the effect of substantial changes (50% to 150% - encompassing experimental disparity) to these key parameters as indicated in Figure 3C. Because the intracellular chloride concentration in the simulation, 4.5 mM, was the same as that used in the intracellular electrode during experiments by Smirnov et al. [10] (an already low estimate of chloride concentration), we did not test lower chloride concentrations. GABA_A_ mediated depolarization was surprisingly robust to even large parameter perturbations (Figure 3C). Changes to GABA_A_ conductance and bicarbonate permeability exhibited the largest effects - a reduction of GABA_A_ conductance or bicarbonate permeability by 50% drastically reduced the GABA-mediated excitation. This is not surprising – the result indicates that a sufficient number of GABA_A_ receptors need to be simultaneously activated and permeable to depolarizing bicarbonate in order to observe depolarization.

**Figure 4:**
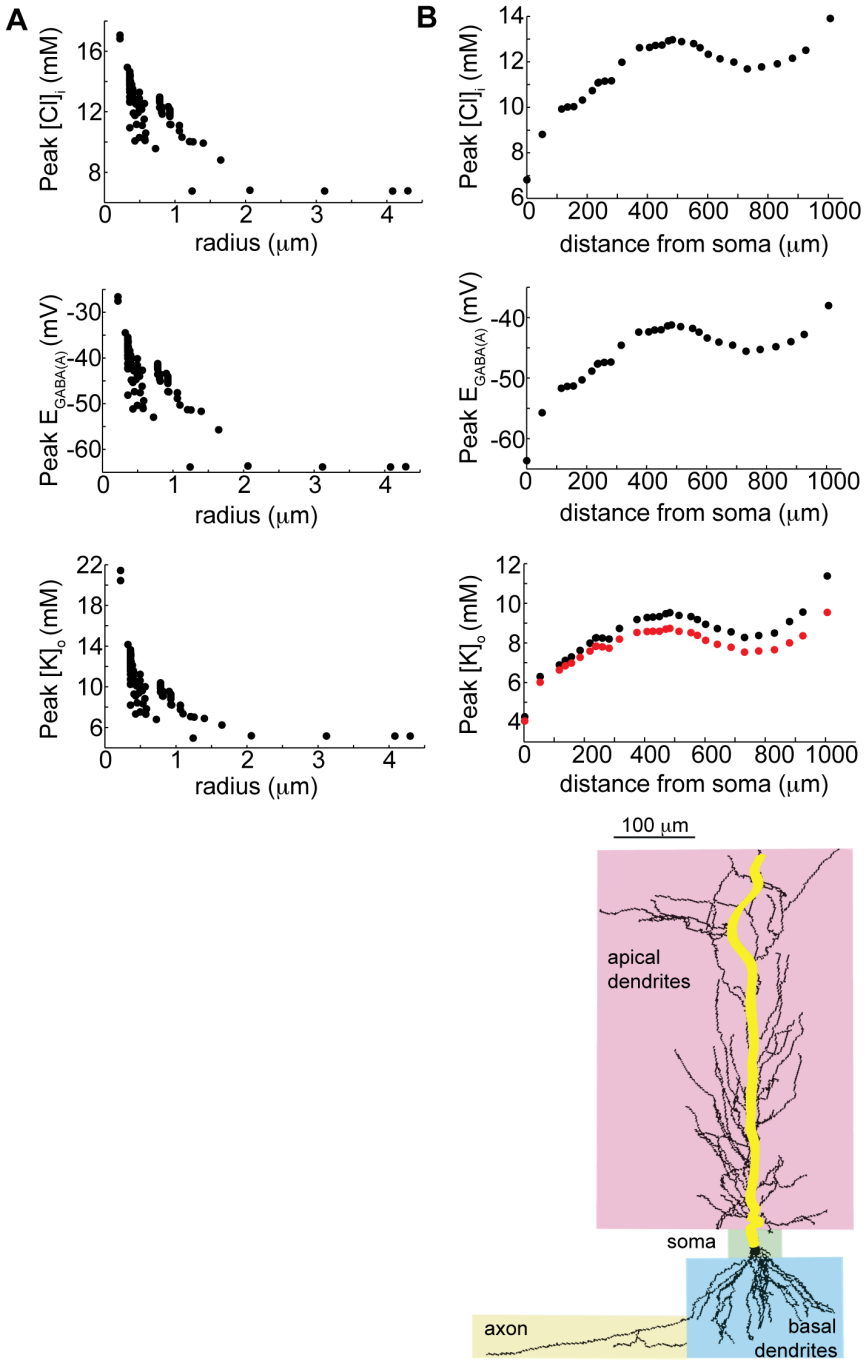
Summary of concentration and GABA_A_ reversal potential changes at various locations. A) The maximum chloride concentration, GABA_A_ reversal potential, and extracellular potassium concentration reached in all somatic and apical dendritic compartments in the model neuron following high-frequency GABA_A_ stimulation, as a function of compartment radius. B) The maximum concentrations and reversal potentials reached along the main apical dendritic tree (highlighted on the image of the neuron) as a function of distance from the soma. Bottom panel: maximum extracellular potassium accumulation in each compartment (black dots), and proportion derived from KCC2 extrusion alone (red dots).

The model also revealed marked heterogeneity of intracellular chloride, E_GABA_, and extracellular potassium along the somato-dendritic tree (Figure 4– yellow line in schematic indicates the path for plotted values). Ion accumulation, as expected, was inversely proportional to the radius of the compartment (Figure 4A) and increased with distance from the soma (Figure 4B). Extracellular potassium accumulation primarily results from extrusion via the KCC2 transporter (Figure 4B, red trace in bottom panel, versus potassium from all sources in black), indicating strong dependence of extracellular potassium transient magnitude on intracellular chloride. The relationship between ion accumulation, dendritic size, and distance from soma was not strictly monotonic, since the distribution of GABA_A_ receptors varies by compartment radius and distance from soma in a non-linear fashion based on the experimental data as detailed in the Methods [32].

### Ionic Mechanisms of GABA_A_ Mediated Depolarization

A transient buildup of extracellular potassium in the pyramidal cell layer following HFS will cause direct depolarization of the cell membrane [9] [5], [10]. But, it is not clear if direct depolarization of the extracellular potassium transient is a sufficient depolarizing source to cause GABA_A_-mediated depolarization.

**Figure 5:**
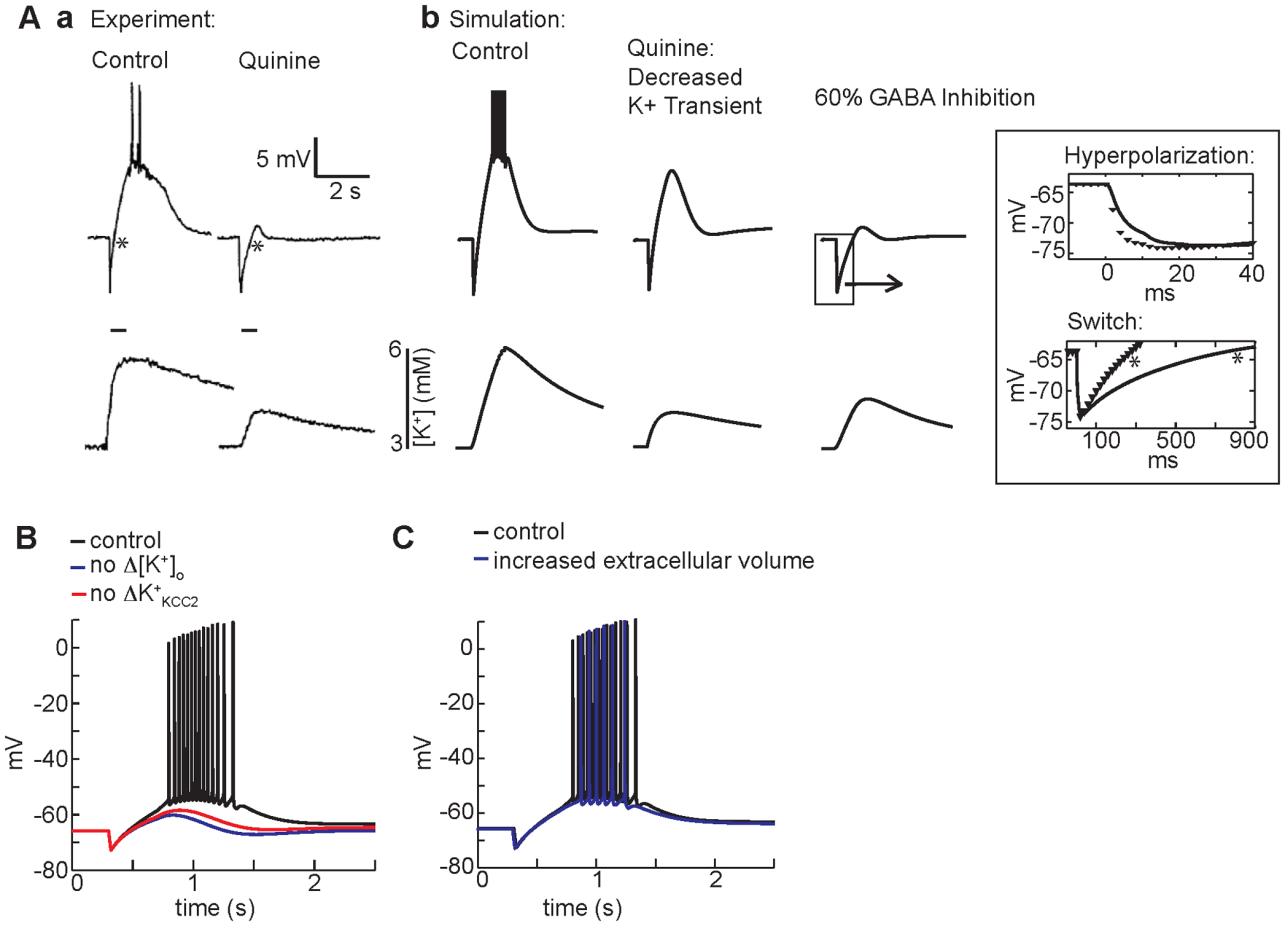
The ionic mechanism of GABA_A_-mediated depolarization. (A) (a) In experiments from Smirnov et al. [10], bath application of quinine blunts the GABA_A_-mediated depolarizing response (top panels show membrane potential measured in the soma) to HFS (a, bottom panels). (b) In the simulation, clamping the extracellular potassium transient to experimental values slightly reduces the amplitude of GABA-mediated depolarization (middle). When effects of quinine on GABA_A_ conductance (60% inhibition of GABA_A_) are included, the simulated voltage traces closely resembles the experimentally observed kinetics of hyperpolarization and diminished depolarization (b, right). The potassium transient is diminished, but not eliminated when GABA_A_ is inhibited - also consistent with the experiment. Inset: magnification of magnitude and kinetics of the hyperpolarization with GABA_A_ inhibition (black line) compared to control (black symbols). In the top panel, slower hyperpolarization occurs because of the decreased magnitude of current elicited by each GABA pulse. Note also the slower switch from hyperpolarization to depolarization in the presence of quinine in experiment (a, right) and simulation (bottom inset) (both marked with *). (B) Complete elimination of the extracellular potassium transient decreases GABA_A_-mediated depolarization (blue line) compared to control (black line). A similar decrease is observed when the potassium transient is unchanged, but the KCC2 transporter is insensitive to the potassium transient (red line). (C) An increase in extracellular volume of the dendritic compartments (22% of intracellular volume) (blue line) compared to control (15% of intracellular volume) (black line) has smaller effects on somatic membrane potential response in the model.

One experiment used quinine, a known potassium channel blocker, to inhibit the potassium transient [10]. A reduction in the amplitude of GABA_A_-mediated depolarization was observed, although the amplitude of the initial GABA hyperpolarization was maintained [10] (Figure 5Aa). These data suggest that quinine eliminates GABA_A_-mediated depolarization by directly acting on the extracellular potassium transient, rather than decreasing GABA_A_ current [10]. However, when we reduced the extracellular potassium transient in the model to match the experimentally recorded transient (by clamping the extracellular potassium to experimental values), there was a slight reduction in the magnitude of GABA_A_-mediated depolarization (4Ab, middle panel), but was much smaller than in the experiment (Figure 5Aa, right). In our model, the reduction in maximum depolarization was less than 4 mV. Thus, in our model, reduction in the extracellular potassium transient alone did not underlie the dramatic reduction in depolarization seen with experimental quinine application.

Recent experimental evidence shows that quinine directly inhibits GABA_A_ receptors [45] with IC_50_ values between 0.4 and 1.6 mM for two different human GABA_A_ receptor subtypes. The experiments by Smirnov et al. [10] applied 0.2–0.5 mM quinine in the bath (IC_50_ values for rat GABA_A_ receptors have not been reported), suggesting that quinine may have resulted in partial inhibition of GABA_A_ receptors. We estimated, given measured IC_50_ values, that the experimental quinine concentration of 0.5 mM would have caused a 60% maximum inhibition of the GABA_A_ current. Simulation of this 60% inhibition of the GABA_A_ receptor by quinine resulted in a voltage timecourse very similar to the experimental recording (Figure 5Ab, right panel). Importantly, the magnitude of hyperpolarization was preserved, despite the pronounced decrease in subsequent depolarization.

Close inspection of the kinetics of the hyperpolarization reveals a clear prolongation in the switch to depolarization that is observed in both the experiment (compare stars in Figure 5Aa and note widening of hyperpolarized voltage segment in the quinine case versus control) and the simulations with GABA_A_ block (Figure 5Ab, right panel and inset). The model predicts that with partial GABA_A_ inhibition, each pulse of GABA results in less current and less chloride accumulation leading to an apparent slowing in the kinetics of the GABA_A_ mediated switch from hyperpolarization to depolarization, though ultimately the same magnitude of hyperpolarization achieved (Figure 5Ab, right panel and inset). Following GABA_A_ inhibition the model also predicts depolarization is slowed and attenuated (compare Figure 5Aa (right) and Figure 5Ab, right panel and inset) and the extracellular potassium transient is decreased but not completely eliminated (Figure 5Ab, bottom right), as observed experimentally (Figure 5Aa, bottom right). Quinine reduced GABA_A_ receptor chloride influx, which then reduced potassium efflux via KCC2, leading to reduced potassium transients.

In the absence of any extracellular potassium transient (Figure 5B blue line) compared to control (black line), attenuation of the GABA_A_-mediated depolarization *is* observed. Elevated extracellular potassium may augment GABA_A_-mediated depolarization either by directly depolarizing the membrane potential or by altering the activity of the KCC2 transporter and, consequently, intracellular chloride and the GABA_A_ reversal potential, which relies on K^+^ gradients to extrude chloride. We used the model to test which effect predominates. When the potassium transient is left fully intact to cause direct membrane depolarization, but the KCC2 transporter is made insensitive to the potassium transient (red line), we observe similar attenuation of GABA_A_-mediated depolarization as under conditions of fixed extracellular potassium. The model predicts that attenuation of GABA_A_-mediated depolarization in the absence of extracellular K^+^ transients results from an increase in KCC2 activity due to a steeper K^+^ gradient and a consequent increase in Cl- extrusion. Increased Cl- extrusion prevents depolarization of the GABA_A_ reversal potential and diminishes GABA_A_-mediated depolarization.

It has been suggested that the extracellular space surrounding the dendrites is less restricted than in the somatic layer [15], but the potassium transients in the dendritic layers of CA1 during GABA-mediated depolarization have not been measured. We increased the size of the extracellular space from 15% of intracellular volume to 22%, as has been reported, at distances greater than 100 µm from the soma [15]. This increase in volume led to decreases in the magnitude of dendritic extracellular potassium transients of approximately 2 mM (depending on the compartment), but GABA_A_-mediated depolarization (Figure 5C) persisted (blue compared to black traces).

### Spatial Heterogeneity Following HFS

**Figure 6:**
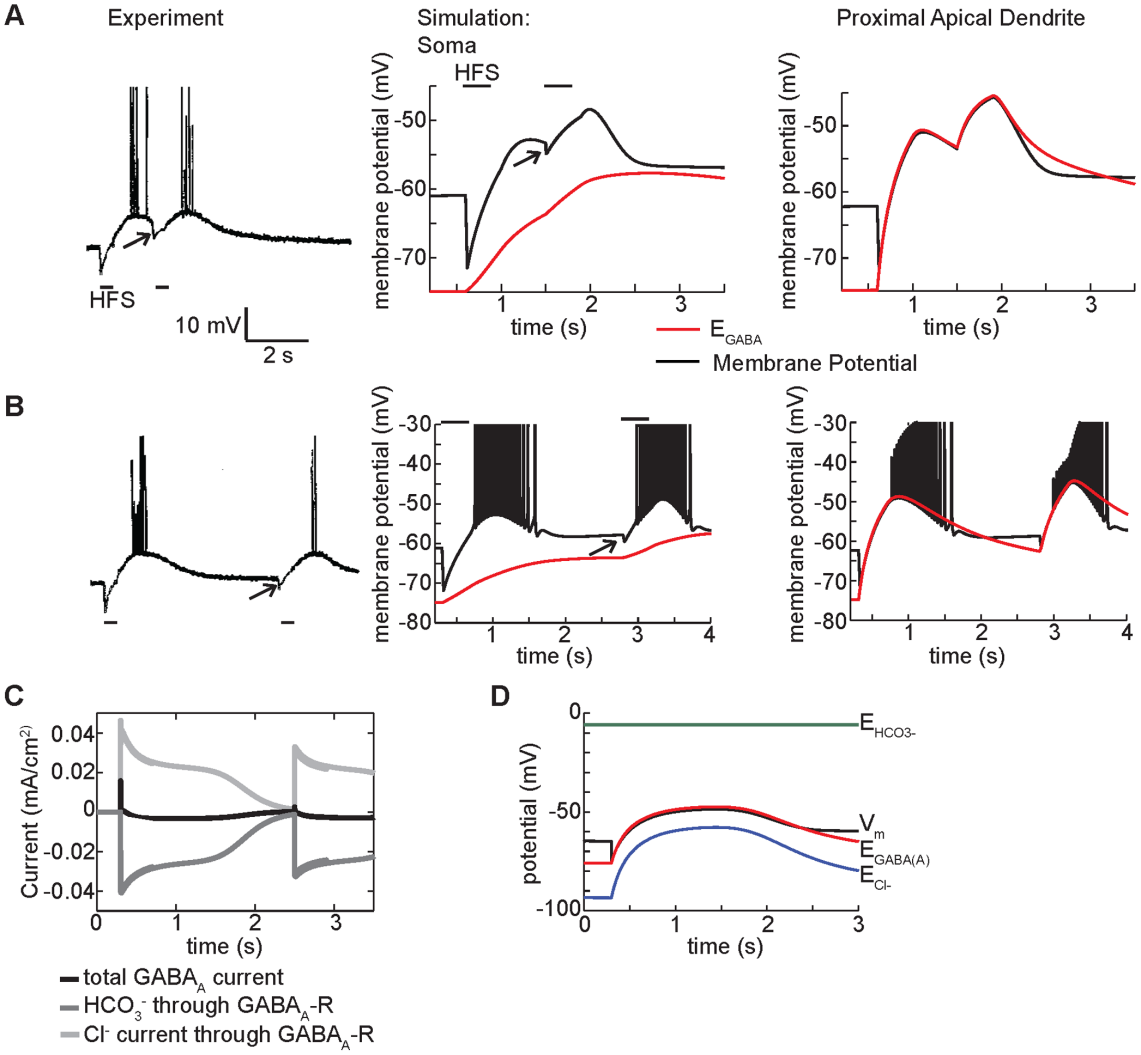
The cellular response to additional GABA_A_ stimulation following high-frequency stimulation. (A) In an experiment by Kaila et al. [9], application of a second identical HFS during the initial falling phase of the GABA_A_-mediated depolarization causes immediate hyperpolarization followed by depolarization. In corresponding simulations (with Na+ channels blocked for clarity) membrane potential (black lines) and GABA_A_ reversal potential (red lines) are shown for a somatic compartment and apical dendritic compartment 150 µm from the soma. As in the experiment, when a second HFS is applied at the start of the falling phase, immediate hyperpolarization is observed in the soma due to the *hyperpolarized* GABA_A_ reversal potential compared to membrane potential. In contrast, immediate depolarization is observed in the dendrite in response to a second HFS because the GABA_A_ reversal potential is *depolarized* compared to membrane potential. (B) When a second HFS is applied following cell recovery from GABA_A_-mediated depolarization, a brief low amplitude hyperpolarization in the experiment, and in the both the somatic and dendritic compartments in the simulation occurs, due to hyperpolarized GABA_A_ reversal potential relative to the membrane potential. The hyperpolarization is short-lived however, owing to the small additional influx of chloride required in the dendritic compartments to push the GABA_A_ reversal potential into the depolarized regime, leading to robust membrane depolarization via bicarbonate current. (C) Total current through the GABA_A_ receptor, and the component chloride and bicarbonate currents. The chloride current dominates initially leading to a fast initial positive total current, which then switches direction as the bicarbonate current dominates. A second subsequent GABA stimulus leads to a repeat of the positive to negative current switch. (D) The chloride reversal potential rises following the high frequency stimulus but does not become depolarized compared to the membrane potential. However, the GABA reversal potential, a weighted average of the chloride and bicarbonate reversal potentials, does become depolarized compared to the membrane potential.

Kaila et al. [9] observed that a second, identical HFS at the peak or decaying phase of GABA_A_ mediated depolarization led to fast hyperpolarization measured in the soma (reproduced in Figure 6A, left). These data show that the GABA_A_ reversal potential must therefore remain hyperpolarized compared to the membrane potential for the duration of the GABA_A_ mediated depolarization and that presumably some other process causes depolarization. The concurrent extracellular potassium transient is a plausible explanation.

The model allowed us to look more closely at the relationship between the GABA_A_ reversal potential and the membrane potential, and revealed that it is essential to examine these processes at different locations in the neuron in order to understand the source of the depolarization. As in the experiment, the model generates hyperpolarization in the somatic membrane potential in response to a second HFS (Figure 6A, middle panel). Also in agreement with the experiment, the GABA_A_ reversal potential is hyperpolarized compared to membrane potential in the soma and proximal dendrites (Figure 6A, middle panel, red line).

However, the model reveals that in the dendrites, the GABA_A_ reversal potential is *depolarized* compared to the membrane potential for the duration of the depolarization (Figure 6A, right panel, red line). The dendritic compartments have GABA_A_ reversal potential up to 20 mV more depolarized than the soma during GABA_A_-mediated depolarization (Figure 4). Thus the model predicts that dendritic depolarization is the mechanism for depolarization of the soma following a second HFS. During the voltage response, the current through the GABA_A_ receptors in the somatic compartment is always hyperpolarizing with a peak magnitude of 0.013 mA/cm^2^, while the axial current from the adjacent dendritic compartment into the somatic compartment quickly becomes depolarizing with a peak magnitude of −0.015 mA/cm^2^.

When a second HFS is applied following the recovery of the somatic membrane potential from GABA_A_-mediated depolarization (Figure 6B), a brief low amplitude hyperpolarization in the experiment (left panel), and in the both the somatic (middle panel) and proximal dendritic (right panel) compartments in the simulation occurs, due to hyperpolarized GABA_A_ reversal potential relative to the membrane potential. The hyperpolarization is short-lived however, because GABA_A_ reversal potential in the dendrites is still substantially depolarized compared to its resting value. Thus, only a small additional influx of chloride is required in the dendritic compartments to push the GABA_A_ reversal potential into the depolarized regime, leading to robust membrane depolarization via bicarbonate current. The dendrites provide a substantial source of depolarizing current to the cell body and whole-cell depolarization ensues.

Finally, the model allows us to understand the relative contributions of chloride and bicarbonate currents through the closer GABA_A_ receptor. The reversal potentials for chloride, bicarbonate, and the GABA_A_ receptor (a weighted average of E_Cl_ and E_HCO3_), reveals that in the dendrites the GABA_A_ reversal potential is depolarized relative to the membrane potential, although the membrane potential is still positive to the reversal potential for chloride (the chloride current component remains inward). The driving force for chloride is nonetheless sufficiently reduced so that chloride current through the GABA_A_ receptor is smaller in magnitude than bicarbonate current, which causes further depolarization (Figure 6C and 6D).

### Implications of GABA-mediated Depolarization for Subsequent Synaptic and Network Activity

It has been suggested that GABA_A_-mediated depolarization following HFS may also facilitate network synchrony by promoting a recurrent excitatory network between pyramidal cells and interneurons receiving collateral excitatory input from the pyramidal cells [6] [46]. The collaterals from CA1 pyramidal cells are limited to the stratum oriens. Interneurons with cell bodies in stratum oriens may synapse on either proximal perisomatic regions of the pyramidal cells or distal regions, depending on the type of interneuron [47] [6] [46]. It has also been suggested that the “tetanized” dendrites may be unable to respond to phasic stimuli [48]. Therefore, we used the model to ask how will a more physiological synaptic input applied subsequently to HFS manifest in the model neuron? Will the neuron response depend on the input location?

**Figure 7:**
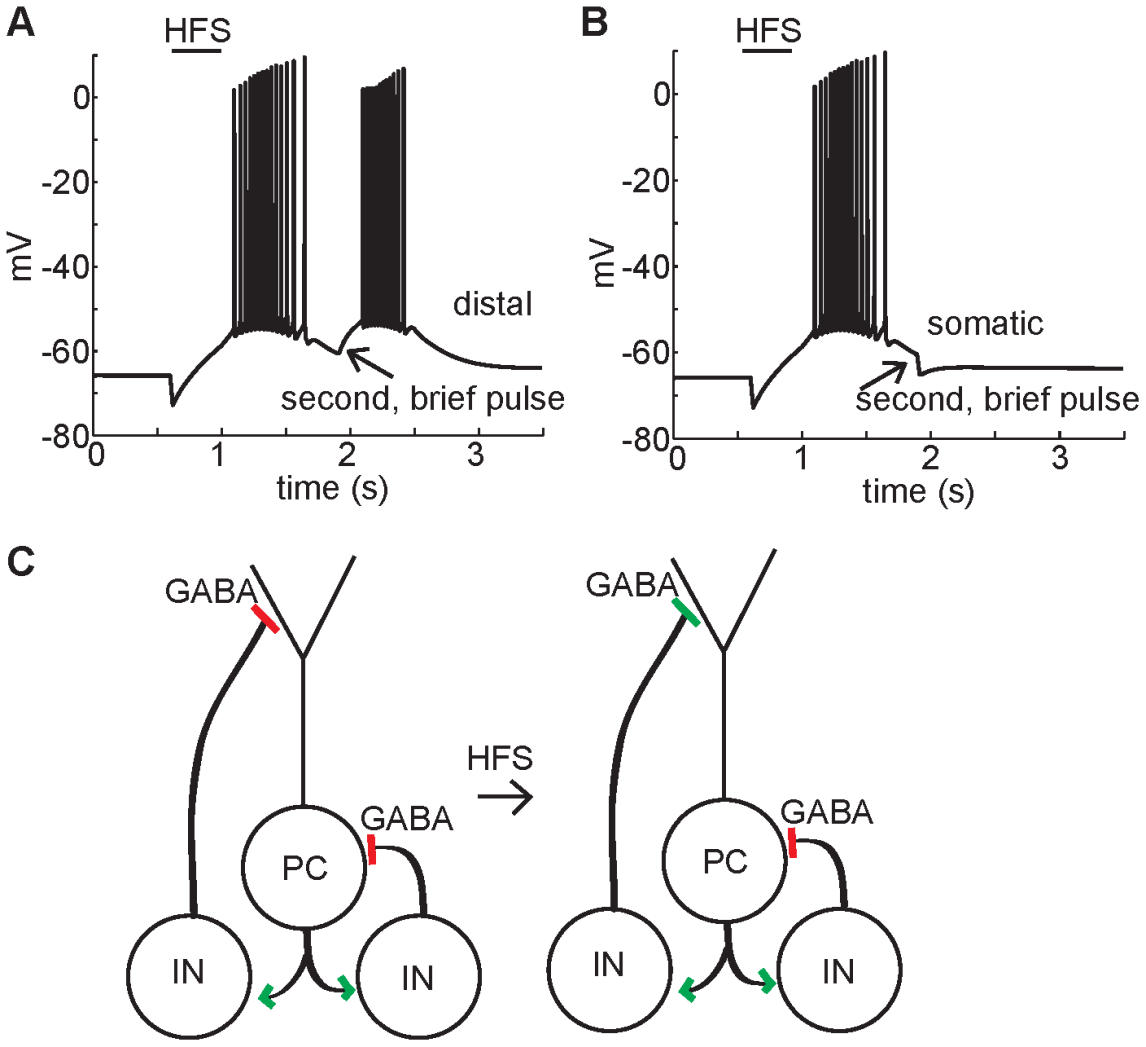
Following high frequency stimulation of GABA_A_, the response of a CA1 pyramidal cell to a physiological stimulus depends on the stimulus location. The somatic membrane potential in response to physiological single pulse of GABA after HFS is shown. (A) A single secondary GABA pulse limited to dendrites with distance 50 to 200 µm from the soma leads to excitation in the soma. (B) A single additional pulse limited to the soma leads to hyperpolarization. (C) The model suggests that interneurons with dendritic targets may have transient excitatory rather than inhibitory feedback onto the pyramidal cell while interneurons targeting the soma will cause inhibitory input.

In our model, a single pulse of GABA (see Methods) applied to the distal dendrites (greater than 200 µm from the soma) following HFS caused an excitatory response manifested as somatic depolarization and action potential firing (Figure 7A). In contrast, a single pulse applied to the perisomatic region following HFS led to hyperpolarization of the soma (Figure 7B). The model thus predicts that ***location matters*** - following HFS, interneurons that synapse on the distal dendrites of the pyramidal cell are likely to establish positive-feedback networks and thus recurrent excitation, while interneurons synapsing on the proximal dendrites and soma are likely to maintain their inhibitory influence (Figure 7C).

**Figure 8:**
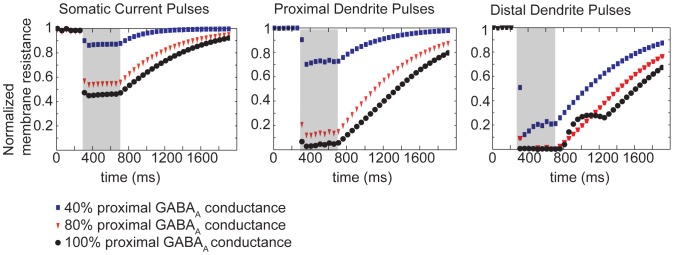
Effects of HFS of GABA_A_ on CA1 membrane resistance. Three levels of GABA_A_ conductances evoke varied degrees of dendritic shunting in response to applied current pulses in the soma (left), proximal dendrite 217 µm from the soma (middle) and distal dendrite 374 µm from the soma (right). Somatic and distal dendritic conductance was fixed at 80% of maximum, while the apical dendritic conductance was 0% (blue symbols), 40% (red symbols), or 80% (black symbols). A 5 ms hyperpolarizing current of 5 nA is applied to the indicated compartment, every 50 ms for two seconds. The response to this pulse is measured in the soma, and the magnitude of the response to each pulse is normalized to the magnitude at baseline. The shaded gray region illustrates the duration of the applied HFS to activate GABA receptors.

The degree of change in membrane potential of the cell in response to flow of ionic current depends on the membrane resistance. When sufficient current is flowing across the cell membrane, for example during GABA-mediated depolarization, the cell is in a “low resistance” state. In this state, additional input, excitatory or inhibitory, may be damped or shunted by the presence of large preexisting currents. Thus, we sought to determine the membrane resistance in the model during GABA-mediated depolarization by applying small hyperpolarizing current pulses at 20 Hz, and measuring changes in the somatic membrane potential. We then compared the baseline response, prior to HFS, to the membrane potential response throughout the GABA-mediated depolarization. An advantage of the model is that we could inject current in the dendrites as well as the soma. The model reveals that, as expected, the membrane resistance is drastically reduced during high-frequency GABA_A_ synapse stimulation (Figure 8). The membrane resistance drops in the soma (left panel), but is much more dramatically reduced in the proximal (middle panel) and especially distal dendrites (right panel). Varying only the proximal apical dendritic GABA_A_ conductance (Figure 8, blue, red and black symbols) leads to large variation in the voltage response to current injection both proximal (in the soma, left panel) and distal (right panel) to the apical dendrites. With maximal GABA_A_ conductance (100%), the membrane in the distal dendrites is “short-circuited” (Figure 8 right panel, black circles) with effectively zero membrane resistance. In this state, any non-GABAergic phasic input has no effect on the membrane potential. Therefore, even though the membrane is depolarized, any additional local input is “shunted”. The membrane resistance in the more proximal dendrites is almost as dramatically reduced to about 5% of the baseline membrane resistance (Figure 8 middle panel, black circles) at 100% GABA_A_ conductance, leading to strong shunting of additional inputs.

The steep decline in membrane resistance during GABA-mediated depolarization supports the experimental observations that during high frequency stimulation of GABAergic interneurons, the post-synaptic pyramidal cell dendritic membranes are effectively clamped to the GABA reversal potential. But it has been suggested that phasic input can still be transmitted in non-tetanized dendrites, and that phasic input transmission recovers quickly in tetanized dendrites following HFS [48]. *In vitro* studies of gamma and beta oscillations suggest that the transmission of phasic input to non-tetanized dendrites and to recovering dendrites plays an important role in shaping network activity following HFS [48].

**Figure 9:**
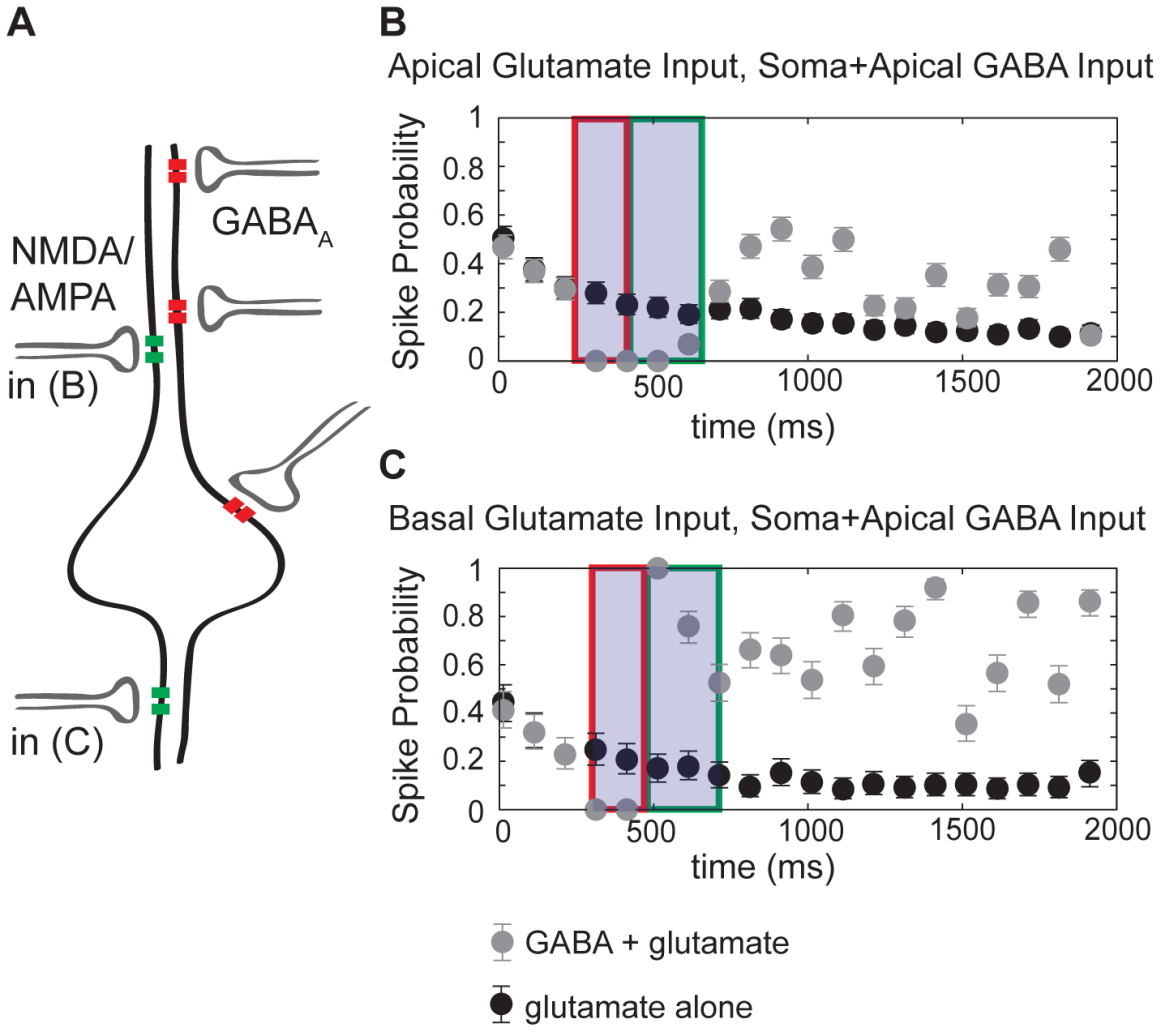
The net effect of interaction between glutamatergic and GABAergic signaling depends on location of glutamatergic input. (A) Schematic indicating location of synaptic inputs in simulations. 10 Hz glutamatergic pulses were applied either to proximal apical dendrites (in B) or basal dendrites (in C). A noisy input current was applied to a somatic compartment so that the probability of spiking in response to a glutamate pulse was less than 50%. For both (B) and (C), HFS of GABA receptors was applied to the soma and apical dendrites (for the duration indicated by the shaded box in B and C). (B) and (C) The probability of action potential firing within 10 ms of a glutamate pulse with GABA (gray circles) and without GABA (black circles) is plotted for 500 trials each, with the error bars representing the 95% confidence interval. During the initial fast hyperpolarization (region outlined by red boxes), HFS and consequent GABA activation completely inhibited action potential firing independently of glutamate input (grey circles). HFS resulting in GABA activation inhibits the response to apical glutamatergic input throughout its course (B), whereas the response to basal glutamatergic input is enhanced (C) (region outlined by green boxes). The slow depolarization following 400 ms of HFS enhanced the response to basal glumatergic input (C) more than apical glutamatergic input (B).

We therefore looked at the model neuron’s response to phasic input, both in the tetanized and non-tetanized dendrites, during HFS and the subsequent GABA-mediated depolarization as depicted schematically in Figure 9A. We injected a noisy current to the pyramidal cell soma so that the probability of action potential firing in response to 10 Hz glutamate pulses applied to either the proximal apical or basal dendrites was below 50%, in the absence of any GABAergic input (Figure 9B,C, black circles). We then compared spiking probabilities induced by glutamate pulses alone to the spiking probabilities in the presence of glutamate pulses and GABA-mediated depolarization induced by HFS.

In the case where the glutamate pulses were applied to the proximal apical dendrites (the site of GABA stimulation), spiking probability dropped throughout the course of 400 ms long HFS of GABA receptors (Figure 3–4B gray circles versus black circles). However, within 10 ms of the termination of HFS, the probability of spiking had recovered and was enhanced throughout the GABA-mediated depolarization.

In contrast, in the case where the glutamate pulses were applied to the basal dendrites (the GABA stimulus was limited to the soma and apical dendrites), spiking probability was *enhanced* during the application of HFS (Figure 3–4C, gray circles versus black circles in region highlighted by the green box). This model prediction is consistent with experimental observations of shunting inhibition [49]. Shunting inhibition is only effective locally, where a short circuit is created in an isopotential region of membrane where the membrane potential becomes clamped to the reversal potential of open channels. However, if the reversal potential of these shunting channels is depolarized compared to resting membrane potential, then farther from the location of the channels, channels underlying the shunting inhibition phenomenon provide a source of axial depolarizing current. Therefore, local GABA shunting inhibition, which diminishes the effect of local glutamatergic input, can actually enhance distal glutamatergic input [49]. This is exactly what is the computational model predicts (Figure 9).

### KCC2 and GABA Depolarization

It has been widely proposed that pathological reductions in KCC2, and the consequent reduction of chloride extrusion, is a likely mechanism of hyperexcitability in the setting of diverse ailments including anxiety, neuropathic pain, and epilepsy [50]–[52] [2] [53]–[55] [3]. However, the recent study by Viitanen et al. [5] suggested that because KCC2 also determines extracellular potassium accumulation, *increased* KCC2 activity might promote excitability. Indeed, KCC2 transporter activity has two competing effects on the membrane potential. On the one hand, maintenance of E_GABA_ following GABA_A_ stimulation depends on KCC2 activity to extrude chloride from the intracellular space. In this manner, KCC2 activity promotes hyperpolarization by maintaining a hyperpolarized GABA reversal potential. On the other hand, KCC2 chloride transport is coupled to potassium transport out of the cell, leading to increased extracellular potassium, which directly depolarizes the membrane.

**Figure 10:**
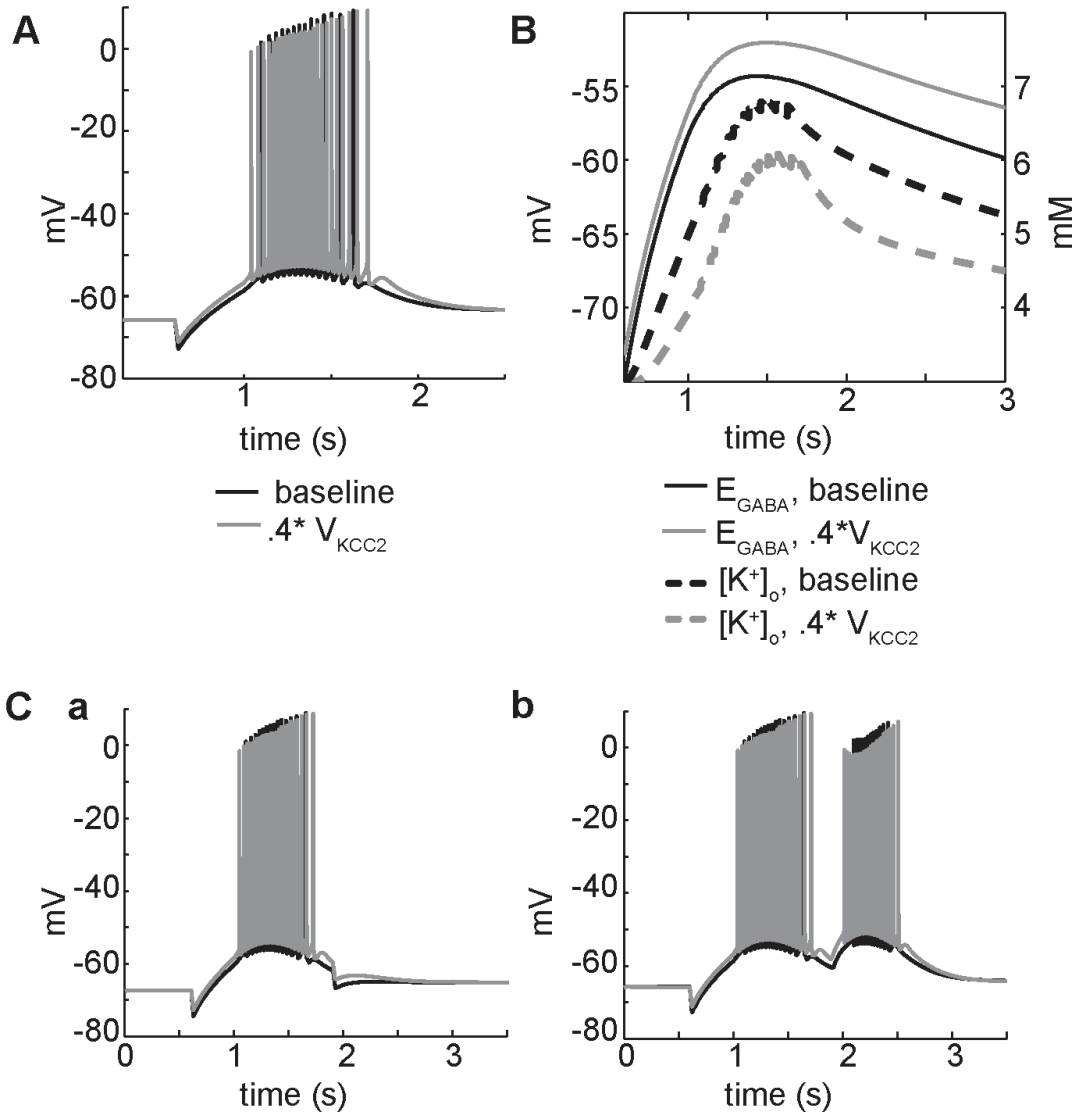
Block of the neuronal K+-Cl– cotransporter (KCC2) promotes GABA_A_-mediated depolarization via effects on GABA_A_ reversal potential. (A) Somatic membrane potential trace in response to HFS for control (black line) and 60% decrease in KCC2 transport (gray line). (B) In a dendritic compartment adjacent to the soma, a decrease in KCC2 leads to increased depolarization of E_GABA(A)_ (solid lines), and a decrease in [K^+^]_o_ (dashed lines). (C) The response to a single GABA pulse following the high frequency stimulus, with 60% KCC2 inhibition compared to control. (a) Somatic pulses with reduced KCC2 are still hyperpolarizing, but dendritic pulses (b) lead to increased depolarization and higher frequency firing (grey) as compared to control (black).

We used the computational model to test which of these mechanisms dominates in the setting of GABA_A_-mediated depolarization. Inhibition of KCC2 activity in the model neuron increases and prolongs GABA-mediated depolarization (Figure 10A, grey line indicates reduced KCC2 activity by 60%- on the order of diminished KCC2 expression in epileptic tissue [51]). The decrease in KCC2 activity leads to prolonged elevation of the GABA reversal potential (Figure 10B, grey lines with KCC2 inhibition versus black lines). Prolonged elevation of the GABA reversal potential alters the cellular response to subsequent GABA pulses - when two low frequency (10 Hz) pulses are applied to the soma, current through the GABA_A_ receptor is still hyperpolarizing as in the control case and is sufficient to inhibit action potential firing (Figure 10Ca). However, when the pulses are applied to the tetanized dendrites, the subsequent excitation under decreased KCC2 transport is stronger and causes higher frequency firing as compared to the control (Figure 10Cb compare grey line during KCC2 inhibition versus black line control). This strong subsequent excitation in the dendrites supports previous suggestions that pathological decreases in KCC2 activity promote hyperexcitability by decreasing neuronal chloride extrusion capacity [51] [53] [56].

## Discussion

Here, we present a computational model of a CA1 pyramidal cell that accounts for ionic homeostatic mechanisms critical to maintain chloride and potassium gradients in CA1 pyramidal neurons. The model reproduces basic electrophysiological features of neurons as measured experimentally, and key to our study, recapitulates paradoxical depolarization observed with high frequency GABAergic stimulation. Simulations suggest plausible ionic mechanisms contributing to GABA_A_-mediated depolarization in CA1 pyramidal cells. Our findings also point to the potential for GABA_A_ activity to cause recurrent excitation in a location-dependent manner, with somatic inhibition likely to be sustained. Finally, our model suggests that the primary impact of decreased KCC2 activity in a single pyramidal cell is increased chloride accumulation and consequent increase in GABA_A_ depolarization, rather than a reduction in extracellular potassium accumulation.

### Intracellular Chloride Buildup Causes GABA_A_– mediated Depolarization

We simulated high-frequency GABA stimulation that has been shown to cause paradoxical excitatory depolarization in post-synaptic CA1 pyramidal neurons. Simulations suggest that GABA_A_-mediated depolarization is primarily caused by chloride accumulation through the GABA_A_ receptor in small dendritic compartments. These findings support experimental data that point to depolarizing bicarbonate current through the GABA_A_ receptor as the mechanistic culprit [4], [7] [8]. Model simulations reproduce hyperpolarization in response to a second stimulus in the ***soma***, however, they predict strong depolarization of E_GABA(A)_ in ***distal dendrites***. The model simulations suggest strong depolarization mediated by GABA_A_ current in the distal dendrites is the source of depolarizing current flowing to the soma (Figure 6).

Although extracellular potassium transients were not predicted to cause substantial membrane depolarization in an isolated post-synaptic neuron, extracellular potassium nonetheless augments GABA_A_-mediated depolarization. We used the model to separate the role of potassium in direct depolarization and the effect of extracellular potassium on chloride transport. Model simulations support the suggestion by Staley and Proctor [7] that the primary influence of extracellular potassium on GABA-mediated depolarization in an isolated neuron is by altering chloride transport via KCC2 (Figure 5).

Our results, do not however, rule out the possible contribution of extracellular potassium transients in sustaining network synchrony following GABA_A_-mediated depolarization. Extracellular potassium dynamics have long been implicated in neurological disease, especially seizure disorders [51], [53], [57]. In our study, we have modeled the response of a single post-synaptic pyramidal cell to a barrage of GABA activity. The resultant depolarization may act as a trigger to a potentially disastrous cascade that results in persistent pathological synchronous bursting in a network of neurons. And, although our model simulations suggest that the “trigger” event is primarily attributable to dendritic depolarization resulting from strong depolarization of E_GABA(A)_, extracellular potassium may nevertheless be required for *persistent* network bursting [5]. Recently, models have been developed that incorporate extracellular potassium dynamics in neurons and neuronal networks. These models have proven useful to aid in interpretation of conflicting experimental data and shed light on the role of potassium mechanisms in seizure activity [58], [59] [60]–[61]. Our simulations suggest that the additional role of potassium homeostasis–its critical influence on chloride transport and inhibitory synaptic strength–should also be accounted for in our understanding of neural network behavior.

### Response to GABA_A_ Depends on Synapse Location

Following high-frequency stimulation with intact glutamatergic and GABAergic synaptic transmission, Isomura et al. [46] found that the fast-spiking interneurons receiving glutamatergic input from pyramidal cells exhibit long-term seizure-like synchrony with pyramidal cells. Some types of interneurons including basket cells synapse onto the perisomatic region of the pyramidal cell, while bistratified and oriens-lacunosum moleculare (O-LM) interneurons synapse onto more distal dendrites [47]. Our model suggests that the synapses made by these GABAergic interneurons will not be uniformly depolarizing, rather their influence will be determined by the location of the GABA synapses (Figure 7). Simulations predict that secondary stimuli to the distal dendrites will cause depolarization, while repetitive stimuli to the soma and perisomatic region will lead to hyperpolarization.

The implications of the model prediction for network behavior are as follows: Simulations suggest a transient positive-feedback network will be established between the pyramidal cell and bistratified or O-LM cells. A more stable negative-feedback network will occur between the basket cells and the pyramidal cells. Key questions for future studies include: 1) Can the interaction between heterogeneous networks cause seizure-like dynamics seen *in vitro*? 2) Can such heterogeneity underlie the progression from gamma to beta frequency network firing observed in experimental seizure models, as suggested by Vreugdenhill et al. [48]?

Despite the drastic reduction in membrane resistance in postsynaptic CA1 pyramidal cells following a brief application of HFS to GABAergic interneurons ([5]; Figure 8), CA1 cells nonetheless fire action potentials and respond to phasic stimuli. However, our model predicts that the response to both glutamatergic and GABAergic phasic stimuli will be highly variable depending on the location of the input.

Self-sustaining network activity following HFS of GABAergic interneurons is known to be dependent on glutamatergic transmission, yet it is unknown whether it is glutamatergic input to the interneurons or recurrent glutamatergic input to pyramidal cells that is required. Our model simulations predict that during the application of HFS to GABAergic interneurons, only excitatory input to non-tetanized CA1 dendrites will cause phasic excitation, while excitatory input to tetanized CA1 dendrites will be shunted. Immediately following a bout of HFS to GABAergic interneurons, excitatory input anywhere along the CA1 dendritic tree will be enhanced (Figure 9). What role this biphasic interaction between GABA and glutamatergic input has on network activity will need to be the subject of future studies.

### Chloride Homeostatic Mechanisms as Therapeutic Targets

Changes in KCC2 density have been observed in a variety of human and animal models of epilepsy, neuronal trauma, and stress [50]–[52] [2] [53]–[55] [3]. A recent study has suggested that loss of KCC2 may be a compensatory response leading to reduced extracellular potassium accumulation [5]. Although our model is consistent with recent experimental observations that suggest KCC2 as the source of extracellular potassium accumulation in response to GABA_A_-mediated chloride accumulation, simulations predict that loss of KCC2 actually increases firing and prolongs GABA_A_-mediated depolarization in single cells (Figure 8). The model also predicts that altered chloride homeostasis due to increased NKCC1 expression [51] [62] [53] [55], or enhanced GABA_A_ activity as seen in experimental febrile seizures [63] may promote GABA_A_ mediated hyperexcitability. Future studies should also include a detailed representation of the ClC-2 chloride channel, which has recently been shown to constitute a chloride influx pathway under physiological conditions that can directly regulate neuronal excitability [64].

### Why Use a Model to Study GABA Mediated Depolarization?

The question of which molecular mechanisms underlie GABA_A_-mediated depolarization has been studied experimentally for decades. These experiments have led to confounding results due, in part, to limitations in existing experimental approaches including: 1) Drugs targeting extracellular potassium and KCC2 directly affect GABA_A_ [38], [40]. 2) Hippocampal pyramidal cell geometry is complex, yet recordings are almost uniformly somatic and thus reflect myriad processes occurring throughout the cell – including those in small dendrites that are so difficult to isolate experimentally. The advantage of a detailed biophysical model is that perturbations, processes and mechanisms are easily isolated and can be studied in different cellular locations [65].

The model simulations presented in this study give rise to a set of potentially experimentally testable predictions. One major prediction of our model is the importance of the dendrites in generating the GABA_A_-mediated depolarization. Our model predicts that functional removal of dendrites beyond the perisomatic region will eliminate the depolarization. This could be tested experimentally by dissociating apical and basal dendrites at various points along the dendritic tree [66] to test if they are required for GABA_A_-mediated depolarization. Alternately, localized application of bicuculline would help to elucidate the role of synapses in different locations. This approach would also allow for testing the model prediction that inhibition of GABA_A_ in the perisomatic region enhances depolarization.

The model predicts that a secondary stimulus from a basket cell, projecting onto the perisomatic region will be inhibitory while a secondary stimulus from a bistratified cell projecting to apical and basal dendrites may promote further excitation. This could potentially be tested experimentally by recordings in a post-synaptic CA1 cell in response to secondary stimulation of specific subtypes of interneurons.

Location dependence of responses may also be relevant to understanding underlying mechanisms of epilepsy. For example, models of epilepsy have shown differential changes in somatic and dendritic GABA synapses [63] [67]. Observed reductions in dendritic synapses with concomitant increases in somatic synapses would be predicted to protect against GABA-mediated depolarization.

Simulations support the claim that decreases in KCC2 transport will lead to increased GABA_A_-mediated depolarization in isolated post-synaptic neurons. In other words, KCC2 blockers would be expected to increase seizure trigger events. As more specific KCC2 blockers are developed, this will be testable experimentally. At present, it would be interesting to determine whether tissue from human temporal lobe epilepsy patients is more susceptible to the seizure-like network activity observed in vitro following tetanic stimulation as compared to normal human tissue, since chloride homeostasis mechanisms have been found to be disrupted in such tissue [1], [51] [53]–[54]. Our model would suggest that pathological specimens would be more susceptible to the GABA_A_-mediated excitation because of the diminished capacity for chloride extrusion. Of course, it would also be critical to consider how elevated extracellular potassium affects other network structures and populations of neurons. The less critical role of increased extracellular potassium in mediating the response in our post-synaptic cell model does not preclude the possibility that reductions in KCC2 may be therapeutic by reducing extracellular potassium sufficiently to prevent persistent network bursting [5]. Further experiments to determine changes occurring in epilepsy, combined with modeling, will help elucidate the role of GABA_A_-mediated depolarization in seizure generation.
