# Correction: Computational Modeling Reveals Dendritic Origins of GABA_A_-Mediated Excitation in CA1 Pyramidal Neurons

**DOI:** 10.1371/annotation/d6af29ee-25c0-4f8e-910e-afe53ef8f963

**Published:** 2013-05-28

**Authors:** Naomi Lewin, Emre Aksay, Colleen E. Clancy

Figure 2C and Figure 3B in this article reproduced images published in Spruston et al. (Reference 68 in the article) and Figure 3B in Viitanen et al. (Reference 5 in the article).

These images are subject to copyright by the journals that published the articles. PLOS ONE requires that all figures and images are published under the Creative Commons Attribution License and as a result, we have removed the previous Figure 2C and Figure 3B from this article and replaced them by original figures generated using the data from the experimental traces reported in the publications, the data were extracted using the program DigitizeIt and plotted in Matlab. 

